# Draft Genome Sequences of 17 French *Clostridium botulinum* Group III Strains

**DOI:** 10.1128/genomeA.01105-15

**Published:** 2015-10-01

**Authors:** Cédric Woudstra, Caroline Le Maréchal, Rozenn Souillard, Marie-Hélène Bayon-Auboyer, Isabelle Mermoud, Denise Desoutter, Patrick Fach

**Affiliations:** a Université Paris-Est, Anses, Food Safety Laboratory, Platform IdentyPath, Maisons-Alfort, France; b Anses, UEB, Ploufragan-Plouzané Laboratory, Hygiene and Quality of Poultry and Pig Products Unit, Ploufragan, France; c Anses, UEB, Ploufragan-Plouzané Laboratory, Avian and Rabbit Epidemiology and Welfare Unit, Ploufragan, France; d LABOCEA, Ploufragan, France; e LNC, Veterinary Diagnostic Laboratory, Paita, New Caledonia

## Abstract

Animal botulism is mainly associated with *Clostridium botulinum* group III strains producing neurotoxin types C, C/D, D, and D/C. In this report, we present the draft genome sequences of fourteen strains of *Clostridium botulinum* producing type C/D and two strains producing type D/C isolated in France, and one strain producing type D/C that originated from New Caledonia.

## GENOME ANNOUNCEMENT

Animal botulism is caused by group III *C. botulinum* strains that produce type C and D toxins, or a chimeric fusion of C and D termed C/D or D/C toxin (1). Animal botulism is considered an emerging disease in Europe, notably in poultry production (2), where it could lead to significant economic losses (3). The actual development of molecular tools allows rapid detection (4) and characterization (5) of the strains involved in an outbreak. We previously showed (4) that animal botulism in Europe is mainly due to mosaic type C/D for avian species, and type D/C for bovine.

Currently fourteen genomes of *Clostridium botulinum* group III are available in the public database: two type C, seven type C/D, three type D, and two type D/C. They originate from different countries but none originate from France.

In order to investigate the epidemiological genetic relationship of strains originating from different geographical areas, we sequenced seventeen strains of *Clostridium botulinum* group III: fourteen type C/D and three type D/C. They all originate from France, except for one type D/C, which was isolated from New Caledonia. Isolates of type C/D originate from chicken, duck, guinea fowl, and turkey, whereas type D/C comes from bovine.

Genomic DNA was extracted from a 48-h culture, incubated at 37°C under anaerobic conditions, in TPGY medium, using the DNeasy blood and tissue kit (Qiagen, Hilden, Germany) according to the manufacturer’s instructions for Gram-positive bacteria, with an additional RNase A (Roche) treatment. Libraries were prepared using the Nextera XT kit (Illumina). Whole-genome sequencing was performed using an Illumina MiSeq platform (Illumina) according to the manufacturer’s instructions. Three MiSeq runs were carried out, two with paired-end 150-nucleotide (nt) reads on MiSeq V2 and V2 microchemistry, another with paired-end 300-nt reads on V3 chemistry. The raw reads were trimmed (minimum length 35 bp, quality score 0.03) and assembled in CLC Genomics Workbench version 7.5.1 by *de novo* assembly (minimum contig length 1,000 bp), producing 75 to 146 contigs (Table 1). The median read depth of the assemblies ranged from 39× for isolate 69285 to 223× for isolate 47295 with *N*_50_ values between 36 kbp and 68 kbp (Table 1). The sequences were annotated with the National Center for Biotechnology Information (NCBI) Prokaryotic Genomes Automatic Annotation Pipeline (PGAAP) at http://www.ncbi.nlm.nih.gov/genome/annotation_prok.

**TABLE 1:** NCBI accession numbers and assembly metrics of *Clostridium botulinum* group III draft genomes

Isolate	Origin	Year	BoNT^a^ type	No. of contigs	Genome size (Mbp)	G+C %	*N*_50_ (Kbp)	Median read depth (×)	No. of coding sequences (per PGAAP)	GenBank accession no.
12LNRI	Duck	2012	C/D	131	3.00	27.8	42	62	2,768	LGVR00000000
12LNR10	Turkey	2012	C/D	132	3.04	27.9	40	77	2,798	LGVQ00000000
12LNR13	Chicken	2012	C/D	140	3.07	27.9	42	63	2,831	LGVT00000000
29401	Chicken	2008	C/D	112	3.04	27.9	57	123	2,806	LGVP00000000
38028	Chicken	2008	C/D	104	3.12	27.9	48	70	2,861	LGVO00000000
43243	Guinea fowl	2009	C/D	111	3.00	27.9	57	57	2,764	LGVU00000000
48212	Duck	2008	C/D	75	3.00	27.9	68	101	2,763	LGVS00000000
49511	Chicken	2008	C/D	85	3.09	27.9	41	83	2,837	LHYP00000000
50867	Chicken	2008	C/D	134	3.08	27.8	36	90	2,849	LHYQ00000000
55741	Turkey	2008	C/D	103	3.04	27.9	57	50	2,801	LHYR00000000
58272	Chicken	2008	C/D	128	3.07	27.9	40	73	2,845	LHYS00000000
58752	Chicken	2008	C/D	114	2.82	28.0	42	73	2,581	LHYT00000000
69285	Chicken	2008	C/D	146	2.94	28.0	57	39	2,717	LHYU00000000
71840	Chicken	2008	C/D	145	3.00	27.9	40	128	2,764	LHYV00000000
LNC5	Bovine	2013	D/C	83	2.89	28.0	56	81	2,579	LHYW00000000
47295	Bovine	2009	D/C	108	3.18	27.9	51	223	2,899	LHYX00000000
51714	Bovine	2009	D/C	101	3.18	27.9	59	116	2,889	LHYY00000000

a BoNT, botulinum neurotoxin.

The average size of the genomes in this study is 3.09 Mb, 2.82 Mb being the smallest genome size, with an average G+C content of 28.9% (isolate 58752, Table 1), and 3.18 Mb being the largest genome size (isolate 47295 and 51714, Table 1). On average, 2,785 coding sequences were identified in the genomes (Table 1). A detailed report on further analyses of the draft genome sequences will be released in a future publication.

### Nucleotide sequence accession numbers. 

The annotated draft whole-genome sequences of these *Clostridium botulinum* group III strains were deposited in DDBJ/ENA/GenBank under the accession numbers listed in Table 1. The versions described in this paper are the first versions.
